# User involvement in ageing and health research: a survey of researchers’ and older adults’ perspectives

**DOI:** 10.1186/s12961-022-00894-3

**Published:** 2022-09-01

**Authors:** Maya Kylén, Björn Slaug, Oskar Jonsson, Susanne Iwarsson, Steven M. Schmidt

**Affiliations:** Department of Health Sciences, Lund University, HSC Margaretavägen 1 B, 22240 Lund, Sweden

**Keywords:** User involvement, Patient and public involvement, Ageing and health research

## Abstract

**Background:**

User involvement in research has rapidly increased and is often a precondition to obtain research funding. Benefits such as effectiveness and increased relevance of research are described in the literature, but the evidence to support this is weak. Little is known about ageing and health researchers’ experiences and perspectives towards user involvement in research, and their attitudes towards user involvement compared to the attitudes of the users involved are largely unknown. To examine researchers’ experiences and perspectives of user involvement in research on ageing and health, and to compare their attitudes towards user involvement to the attitudes of older adults in the general population.

**Methods:**

A panel study survey was used to elicit responses from researchers in ageing and health as well as from older adults (aged 60 years and older). The researcher sample (*N* = 64) completed the survey online, while the older adult sample (*N* = 881) could choose among three different options to complete the survey (online, paper format, telephone). A professional survey company collected the data. Descriptive statistics, exploratory comparisons and descriptive qualitative content analysis were used to analyse the data.

**Results:**

More than half (58%) of the researchers had previous experience of involving different categories of users in a wide range of research activities. The most frequent motivation for involving users was to ensure that the research produced is relevant to the target population. A majority (86%) reported benefits, and more than half (59%) described challenges. Differences in attitudes were found between researchers and older adults in the general population.

**Conclusions:**

Ageing and health researchers involve users in their research to improve quality and ensure relevance, but there is no consensus among them whether users should be involved in publicly funded research. While several challenges were identified, training, institutional support and resources from funders could alleviate many of these. Findings reveal significant differences in attitudes between older adults in the general population and researchers. Further research with comparable larger samples is needed to confirm and understand the possible consequences such controversy might have and how to solve them.

IRRID (International Registered Report Identifier): RR2-10.2196/17759.

## Background

Working in partnership with knowledge users in research has the potential to generate improved identification of research priorities, framing of research questions and credibility of results [1–3]. As a consequence, the likelihood of influencing policy and practice may increase [4]. Hence, many research funders now encourage researchers to involve users. For example, the European Commission promotes user involvement through research policy formulation, provision of direct support, and a more objective-driven and ambitious partnership approach [5].

Despite strong political underpinnings, the evidence base to support the impact of user involvement is weak and unreliable [6–8]. Thus, researchers are encouraged to involve users in their research to enhance research quality and impact. However, user involvement in research challenges many of the values and beliefs researchers hold (e.g. ethical and/or political issues, consequences of involvement) [9], but these concerns seem to be overlooked by policy-makers and research sponsors [10]. Specific challenges for ageing and health researchers include working with users with diverse capabilities, resources and needs [11]. Ageing and health researchers’ beliefs and behaviours are fundamental to whether, how and at what stages user involvement takes place. Understanding their experiences and perspectives is thus important to gain a better understanding of how and in what contexts user involvement works.

### Who are the users?

The users in this study are older adults in the general population. They are seen as “knowledge users”, a word put forward by WHO [12] to capture the many different categories of users (e.g. patients, advocacy groups, older adults, health and social care services, other professionals and policy-makers) who are interested in or benefit from ageing and health research results directly or indirectly. Seen from this perspective, knowledge users are those who can identify a problem and implement research recommendations [13]. User involvement in research refers to the inclusion of users as active partners in different phases of the research process, which means that the research is conducted with or by users rather than to, about or for them [14].

### Benefits and challenges of involving users in research on ageing and health

The fundamental motive for involving users in research is that it enables reflection on user needs and improves research design, recruitment of participants and research quality through all stages of the research process [6]. Other commonly cited arguments for why users should be involved concern political and moral aspects, for example, that members of the public have the right to take part and influence publicly funded research [15] and that involvement may increase the use of research to shape policy and practice [16]. Beyond the positive impacts on research quality and societal change, user involvement can empower the individuals involved [17], enhance their self-confidence and allow them to develop new skills [18].

Population ageing challenges society in relation to the provision of healthcare and social services systems, person-centred care facilities and age-friendly environments designed to support active and healthy ageing [19, 20] that are complex in nature and require new solutions. While user involvement in research is being advocated to address such challenges, user involvement on complex problems requires a different approach than when problems are clearly described and easily solvable [4]. Knowledge synthesis gained from involving several different categories of users in the research process is thus crucial, but no doubt a challenging task for researchers. Additionally, involving older adults in the research process can be challenging and context-dependent [21], which is complicated by the heterogeneous nature of the older segment of the population (i.e. varied capabilities, resources, experiences, expectations, needs). Poor health and limited mobility among older adults with age related conditions put demands on researchers to carefully consider the location of involvement activities, communication strategies and how to build trust and enhance self-confidence [22]. In addition, even though frail older adults can provide important knowledge and perspectives [11], they can be hard to reach or may be excluded due to stigma that devalues their abilities to contribute [14]. Also, recent research shows that if ageing and health researchers do not consider factors such as health status, age and digital skills when they involve older adults in research, this mechanism may in turn deepen already existing inequalities among older adults [23].

### Researchers’ attitudes

Attitudes among researchers towards user involvement in research deserve to be explored, yet research has primarily focused on patients and clinicians [24] rather than researchers. Few studies focus explicitly on researchers’ experiences of involving users [25], and whether there has been a shift in attitudes following the recent policy requirement to involve users among researchers is not well understood [26]. Despite acknowledging potential benefits of user involvement, researchers may feel reluctant to change, as their knowledge may be challenged [27]. For example, Boaz and colleagues interviewed biomedical researchers and found that the vast majority were reluctant to involve users, as such an approach involves the idea of sharing power [26].

Among the small number of studies focusing explicitly on researchers, Boylan and colleagues [28] interviewed 36 health researchers to explore their experiences and attitudes regarding user involvement in research. They found that user involvement is a complex task and reported a mix including positive, cynical and ambivalent attitudes. They also found that career stage and gender influenced the researchers’ experiences of user involvement activities and that user involvement activities may be facilitated by support from senior colleagues [28]. A survey conducted by the Swedish nonprofit organization Vetenskap & Allmänhet (VA [Public & Science]) showed that four out of 10 of the 3699 responding researchers had previous experience of involving users in research, and user involvement was more common in certain disciplines (e.g. social sciences) [29]. The findings reveal that knowledge about user involvement is more common among senior researchers, and researchers in the natural sciences tend to have more positive attitudes than those in the arts and humanities [29]. Turning to research on attitudes towards user involvement among older adults in the general population, both benefits (e.g. personal development) [30] and challenges (e.g. tokenism) [31] have been described. However, whether there are differences or similarities between researchers' and the general populations’ attitudes towards user involvement remains to be explored.

To build capacity for the future and create more favourable conditions for involving different categories of users in research, further exploration of experiences and attitudes towards user involvement from ageing and health researchers’ points of view is needed.

The overarching aim of the present study was to describe researchers’ experiences of user involvement in research on ageing and health and compare researchers’ and older adults’ attitudes towards user involvement. The following research questions guided the analysis:

1. What previous experiences of involving users do researchers have?
2. What motivates senior and junior researchers with previous experiences of user involvement to involve users in their research?
3. What benefits and challenges are perceived by researchers with previous experience of user involvement?
4. How do the researchers’ attitudes compare to the attitudes of older adults in the general population?

## Methods

The present cross-sectional study is based on the first data collection from a panel study conducted among researchers in ageing and health and older adults (aged 60 years and older), in Sweden. The surveys for the researchers and older adults were based on the same comparable core questions but modified to fit the perspective of the respective group. An overview of the panel study samples, methods and examples of survey questions are presented in the study protocol [32]. A professional survey company was commissioned for the data collection. The panel study is part of the UserAge programme [33]. UserAge has had user involvement from the conceptualization stage throughout the programme providing input on all aspects of the empirical studies. It was approved by the ethical board in Lund (no. 2018/986). Participation was voluntary, all participants gave written or oral informed consent.

### Participants and procedures

The researchers were identified and recruited from partner universities affiliated to the Swedish National Graduate School for Competitive Science on Ageing and Health (SWEAH) and the Swedish Gerontological Society (SGS). In total, 210 people affiliated to these networks were contacted by email with an invitation to participate in the online survey in English. To be eligible, researchers had to have experience with research on ageing and health.

The older adult sample was drawn from the Swedish state personal address register; invitation letters were mailed to 3319 people, with instructions on how to complete the survey (in Swedish) via telephone, online or on paper. Potential participants who had not completed the survey online or declined participation were contacted by phone after 2 weeks and reminded about the various options to complete the survey. The mean age of the participants in the older adult sample (*N* = 881, 29% response rate) was 72.2 (SD = 7.29) years. There were slightly more women (52.9%, *n* = 462) than men, and most perceived their health as good to excellent (73%); 3% rated their health as bad. While a majority (58%) of the older adults had previously been study participants in research, less than one sixth (15%) had previous experience of active involvement as defined in this study. Details regarding the older adults are presented elsewhere [34].

### Survey questionnaire

The survey questionnaire for the older adults was constructed based on relevant literature [35–37] and expertise in the research team. It contained questions on demographics and attitudes towards user involvement in research. To refine the survey for content, time to complete, readability and understandability for the older adults, we engaged a face-to-face user forum with user representatives (i.e. older adults from the general population, representatives from interest organizations) to provide their input during the development phase [32]. Prior to the data collection, a pilot study was conducted.

The survey questionnaire for the researchers was based on a set of comparable core questions and feedback from UserAge researchers. Researchers with previous experience of involving users were asked to select what categories of users (eight response options) had been involved in their research and in what specific activities (13 response options). Benefits and challenges were captured with yes/no questions. If yes, researchers were asked to provide free-text examples of why involving users made a difference or what challenges they had encountered. Motivations for involving users in the research process were captured by a checklist containing nine predefined items (e.g. to improve the design of the study/methodology) and the possibility to add free-text motivations. Questions were also included about the researcher characteristics: age, sex, career stage and disciplinary background.

To capture attitudes about user involvement in research on ageing and health, participants in both samples rated their level of agreement on seven statements (e.g. “People who are affected by research have a right to have input on what and how research is undertaken”) on a four-point scale, ranging from strongly disagree to strongly agree.

### Data analysis

To give an overview of the researcher sample and their previous experiences of involving users, we used descriptive statistics. Among researchers with previous experience of involving users, motivations for involvement are described among senior and junior researchers.

To analyse the data reflecting perceived benefits and challenges expressed by researchers with previous experience of user involvement, we used descriptive content analysis [38]. To reach consensus and validate the categorization of the free-text responses, three researchers (MK, OJ, BS) were involved in the analysis regarding the benefits, and two of them (OJ, BJ) regarding the challenges.

Differences in attitudes between the researcher and the older adult samples were tested with the Mann–Whitney *U*-test. All analyses were performed in SPSS version 26 statistical software [39]. Two-sided *P*-values of < 0.05 served as the overall indicator of statistical significance and were adjusted with the Bonferroni correction for multiple comparisons (*P* < 0.007).

## Results

### Participant characteristics and previous experiences of user involvement

**Fig. 1:**
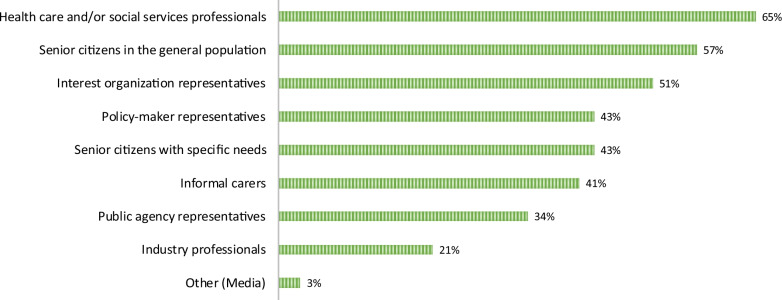
The different categories of users that researchers with previous experience had involved in their research projects, *n* = 37

In total, 64 ageing and health researchers (31% response rate; 73% women; mean age = 47.4 years, SD = 14.4) completed the online survey. There were more junior (67%) than senior researchers (33%). More than half (58%) had previous experience of involving users in their research on ageing and health. See Table 1 for characteristics of the researcher sample. Among ageing and health researchers with previous experience of involving users (*n* = 37), the most common category of user involvement was with healthcare and/or social services professionals (65%), while only one researcher reported involving media in their research. See Fig. 1 for all categories of users involved.

**Table 1:** Characteristics of the researcher sample with and without previous experience of user involvement in research on ageing and health, *N* = 64

Characteristics	Total % (*n*)	Previous experience of involving users in research	
		Yes, % (*n*)	No, % (*n*)
Total sample		57.8% (37)	42.2% (27)
Age, mean, (SD)	47.4 SD = 14.4 (64)	48.8 SD = 11.9 (37)	45.4 SD = 17.3 (27)
Sex			
Women	73.4% (47)	53.2% (25)	46.8% (22)
Men	26.6% (17)	70.6% (12)	29.4% (5)
Career stage^a^			
Senior	32.8% (21)	71.4% (15)	28.6% (6)
Junior	67.2% (43)	51.2% (22)	48.8% (21)
Disciplinary^b^ background			
Social science	45.3% (29)	51.7% (15)	48.3% (14)
Medicine	53.1% (34)	64.7% (22)	35.3% (12)
Engineering	4.7% (3)	33.3% (1)	66.7% (2)

^a^Associate professor yes/no cutoff. ^b^Two reported belonging to more than one discipline

**Fig. 2:**
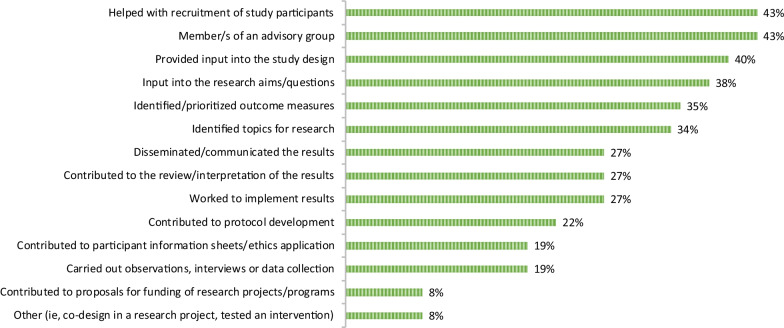
Specific activities researchers with previous experiences had involved users in, *n* = 37

Regarding the specific activities the ageing and health researchers had involved users in, being a member of an advisory group (43%) and helping with recruitment of study participants (43%) were reported most frequently, followed closely by providing input to the study design (40%) and research questions (38%). It was less common that users had been involved in the data collection (19%) or contributed to development of participant information material or ethics applications (19%). See Fig. 2 for all activities.

### Benefits and challenges described by ageing and health researchers with previous experience of involving users

Out of the 37 ageing and health researchers with previous experience of involving users in their research, the vast majority (*n* = 32, 86%) reported benefits and provided examples. Five categories of beneficial consequences of user involvement in research emerged: (1) societal relevance; (2) research quality; (3) methodological resources; (4) improved implementation; (5) improved communication. Examples of benefits for each category of beneficial consequence are presented in Table 2.

**Table 2:** Examples of benefits described by researchers with previous experience of involving users (*n* = 37), categorized into five types of beneficial consequences

Beneficial consequence	Descriptions
Societal relevance	Identify topics/issues from the real world Implementation programme was more focused on the needs the target group had Easier to understand their perspective Relevance of research questions Stay on track on what is really relevant, get different viewpoints Involving carers was of importance to receive knowledge about what they need and what they would want when it comes to online support From a project manager and researcher perspective you might overall focus on developing the product and receive the data that you need BUT when you involve users you might discover that you must consider their life circumstances and what they view as important They represent the target population We could concentrate on those things that mattered most In terms of validity To include their experiences strengthened the relevance of the findings Increase the practice Benefit the public The organization took findings to redesign their intervention approach targeting older people in developing countries
Research quality	To get help with research questions Yes, we understand things better! To enhance the feasibility of the research design I got some good ideas about what questions to ask in interviews and surveys and possible interpretations of results Input on interpretation of the results Relevant and important feedback on our results as well as our plan on how to move forward. By asking questions we could further be clearer in our way of describing the study The trustworthiness of the intervention increased. Greater person-centredness of the instrument/tool we developed It was beneficial to get a deeper understanding about the whole procedure when it comes to co-designing Refined the results Helped to brainstorm solutions to issues encountered during the implementation phase of the research
Methodological resources	Helped to increase the accessibility of the chosen research instruments, interview guides/schedules Helped to design more user-friendly experiments Primarily concerning relevant design of the study Achievement of project goals To get help with interview questions and recruitment of participants We could adapt the methods and design of the devices
Improved implementation	It will make implementation later on easier Implementation of the results in practice Implementation of findings Facilitated implementation of new methods and routines in the organization Possibility for implementation Important input for improving the “product” that was tested/implemented
Improved communication	Helped to disseminate the research findings in their respective networks and in more accessible ways Increase visibility of our research Our research is acknowledged more

More than half (*n* = 22, 59%) of the 37 ageing and health researchers with previous experience of involving users in their research on ageing and health stated that they had experienced challenges. Six categories of challenges emerged: (1) resource demands; (2) recruitment and sustaining participation; (3) representativeness of those involved; (4) involving older adults; (5) involving professionals; and (6) other challenges. Examples of challenges for each category are presented in Table 3.

**Table 3:** Examples of challenges described by researchers with previous experience of involving users (*n* = 37), categorized into six types of challenges

Challenges	Descriptions
Resource demands	Practical and resource demanding The project took longer time Organizational hindrance to get enough time and resources It took a lot of resources (time, people) that could have been used for other tasks Spent some time communicating with the people involved Sufficient funding to pay for transport and other related costs Resources spent on recruiting and training people, where some only did a few interviews. Still, this was not unexpected, and others did more Time consuming to consider and balance different opinions Time
Recruitment and sustaining participation	There is a challenge concerning time as healthcare professionals and managers have difficulties to leave their assignments during working hours Difficult to recruit health professional due to their lack of time. Also, difficult to recruit older adults Lack of time of practitioners, decision-makers and policy-makers to be involved in research for sessions longer than 30 minutes (1 hour maximum) and for more than one session Many decline the invitation to participate Recruitment and sustaining participation over time was a challenge To get enough participants among the users
Representativeness of those involved	Bias in power, do we involve the right users? Who will they represent? Issues of the level/degree of representativeness of the users involved Problems with representation, i.e. who could speak for whom. For example, regarding the voice of people with very complex needs who have difficulties in articulating their views They were not fully representative, so you had to take some notions under consideration before implementing
Involving older adults	Language barriers Concerning the involvement of older citizens one of the challenges has been to ensure that we provide information that is easy to understand Fatigue and short-term memory loss among involved older citizens with advanced, long-standing chronic conditions which involves significant planning and suitably qualified research staff to optimize the enjoyment and desired level of involvement of the older users' having their own agenda for participating in the research Many frail with multiple diseases Another challenge was how to really involve them and encourage them to be involved in “setting the agenda”. It’s easy as a researcher to take too much of the lead Many negotiations Some participants expressed ageistic opinions
Involving professionals	Concerning the involvement of healthcare professionals and managers one of the major challenges has been to ensure that research ethics is ensured There was a need for continuous negotiations between researchers and the professionals to reach consensus. From a researcher perspective it was necessary to make concessions regarding the scientific quality
Other challenges	Lack of knowledge how to involve users Translate findings We were not sure if our findings could be reported without bias by the media

### Motivations for involving users in research on ageing and health

Among ageing and health researchers who had previously involved users (58%, *n* = 37), the most common motivational reason was to ensure that the research was relevant to the target population (76%, *n* = 28). In addition, 62% (*n* = 23) of the ageing and health researchers stated that user involvement was motivated by the quest to strengthen the validity and trustworthiness of the results as well as to strengthen the possibilities for implementation. Improving communication of results with non-academics and society motivated half of these ageing and health researchers (*n* = 18) as well as to improve the design of the study methodology (46%, *n* = 17). It was less common to be motivated by the overall societal encouragement to involve users in research (22%, *n* = 8), and six participants stated that user involvement was important because of ethical priorities and requirements of research funders (16% respectively). In addition, one participant stated that requirements of the hosting institution motivated them to involve users (Table 4).

**Table 4:** Motivations for involving users in research on ageing and health among researchers with previous experience, *n* = 37

What was your motivation for involving users in your research on ageing and health?^a^	Yes, % (*n*)	Career stage^b^, % (*n*)	
		Senior (*n* = 15)	Junior (*n* = 22)
To ensure that research is relevant to target population	75.7% (28)	80% (12)	72.7% (16)
To strengthen the validity and trustworthiness of the results	62.2% (23)	46.7% (7)	72.7% (16)
To strengthen the possibilities for implementation	62.2% (23)	80% (12)	50% (11)
To improve communication of results with non-academics/society	48.6% (18)	60% (9)	40.9% (9)
To improve the design of the study/methodology	45.9% (17)	40% (6)	50% (11)
Inspired by the overall encouragement to involve users in research	21.6% (8)	33.3% (5)	13.6% (3)
Requirement of research funders	16.2% (6)	13.3% (2)	18.2% (4)
Ethical imperative	16.2% (6)	20% (3)	13.6% (3)
Requirement of the hosting institution	2.7% (1)	6.7% (1)	0% (0)
It was imperative to achieve study purpose^b^	2.7% (1)	0% (0)	4.5% (1)

^a^More than one response alternative was possible. ^b^Written as specification of choice “other”

### Attitudes towards user involvement in research on ageing and health

**Fig. 3:**
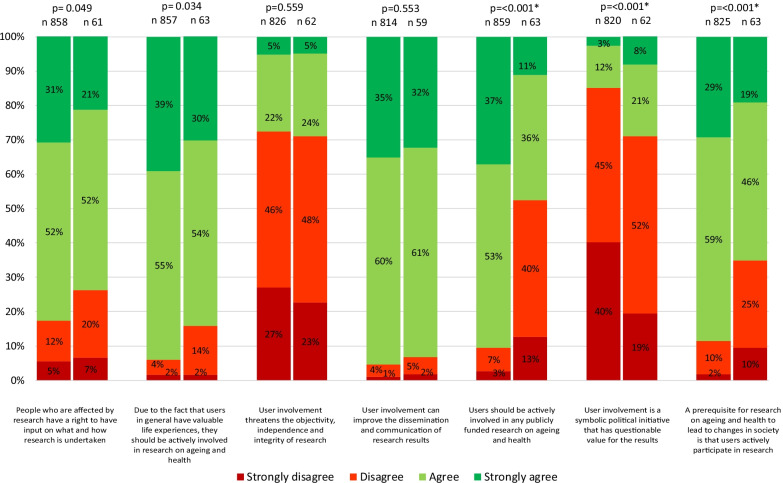
Attitudes among ageing and health researchers compared to the attitudes of older adults. Bars to the right represent the researchers’ attitudes towards user involvement (*N* = 64, *n* varies due to internal missing). Bars to the left represent the older adults in the general population (*N* = 881, *n* varies due to internal missing). The Mann–Whitney *U*-test was used to examine the differences between groups. Two-sided *P*-values of < 0.05 served as the overall indicator of statistical significance and was adjusted with the Bonferroni correction for multiple comparisons to *P* < 0.007, marked with an asterisk (*)

The attitudes towards user involvement in research for both the ageing and health researcher (*N* = 64) and the older adult (*N* = 881) samples are presented in Fig. 3. Turning to the comparison of attitudes between the two samples, a number of significant differences were identified. Older adults to a higher degree than the ageing and health researchers agreed or strongly agreed that the public should be actively involved in any publicly funded research on ageing and health (*P* < 0.001) and the ageing and health researchers to a higher degree agreed or strongly agreed that user involvement is a symbolic political initiative that has questionable value for the results (*P* < 0.001). Furthermore, significantly more (*P* < 0.001) older adults (88%) agreed or strongly agreed that active user involvement is a prerequisite for research leading to changes in society compared to 65% of ageing and health researchers (Fig. 3).

## Discussion

This explorative study focusing on ageing and health researchers’ experiences and attitudes towards user involvement in research reveals interesting results. Not all of the researchers had previous experience of involving users in their research and those who did described that involvement comes with benefits as well as challenges. Older adults in the general population and researchers differed in attitudes towards user involvement. Additionally, we found a lack of consensus among researchers regarding whether users should be actively involved in publicly funded research or whether user involvement can lead to societal change. The study findings increase the understanding of researchers’ beliefs and experiences regarding user involvement in research. Additionally, we highlight similarities and differences in attitudes towards user involvement between researchers and older adults. All together this knowledge is important to generate ideas about how to improve involvement practices in the future.

### Previous experiences and nature of involvement

Considering the encouragement of research funders in Sweden towards public involvement in research and the wide definition of users applied to the researcher survey, it is noteworthy that many researchers had not involved users at all. Perhaps those without user involvement experience are engaged in research for which this is considered less relevant or more challenging, for example, lab-based experimental research or epidemiological research [40]. Even so, we found that slightly more than half had involved users, which is higher than that reported by Barber and colleagues in the United Kingdom [41] and in the VA survey in Sweden [29]. That is, in the United Kingdom study, only 17% of the surveyed 518 researchers had involved users in their health research, and in the VA study, 39% of the 3699 participating researchers had such previous experience. That said, our findings suggest that researchers in ageing and health have more such experiences than researchers in other fields of inquiry. It should, however, be kept in mind that despite major changes to user involvement requirements over the past 15 years, recent studies reporting the prevalence of user involvement in research are sparse [42], making it difficult to summarize the current state and make valid comparisons. In addition, as the implementation of involvement varies internationally, future research should study changes in researchers’ attitudes towards user involvement over time, rather than comparing studies from different countries.

The results reveal that it was more common to involve healthcare and/or social services professionals than older adults in the general population. In a systematic review, Biddle and colleagues [43] found that researchers were more likely to work with patient organizations than with members of the public. This could lead to important perspectives and knowledge about the nature of health and social problems experienced by frail older adults not being heard in research activities [11]. In addition, although less common, the researchers in our study reported that they had engaged public agency representatives or industry professionals in their research on ageing and health. The voices of these categories of users are vastly important for achieving practice and policy change. In the case of complex problems, Bammer [4] emphasized the importance of the breadth of involvement including both users affected and users in a position to achieve change.

Turning to the different activities in which involvement took place, our findings are both similar to and somewhat different from those previously reported. While the participating researchers in our study described previous experience of involving users in all parts of the research process, the view of the researchers in the VA study was that users should be involved primarily at the beginning (e.g. setting research priorities) and at the end (e.g. communicating results), rather than during the research process [29]. Despite these opinions, among the researchers with previous experience in the VA study, the most common user activity was collecting data (18%), followed by having helped with internal or external communication activities (16%) and proposed project ideas (14%). Yet, in comparison with our study, there were fewer response options in the VA study, making the findings hard to directly compare. The survey of health researchers in the United Kingdom [41] found that researchers had most frequently involved users as members of an advisory group and to improve study design and methods, followed by identifying research topics and dissemination of results. These results are comparable to the findings in our study showing, for example, that engaging users as members of an advisory board was a common user involvement activity. Engaging users as members of advisory boards or inviting users to attend regular meetings have been argued to encourage a more active mode of involvement than other methods (e.g. predefined tasks at one or few occasions) [3] and might reflect the ongoing discussion towards research conducted in partnership with greater depth, and empowerment of those involved [4]. Nevertheless, in the context of involving older adults in research, a review of the literature shows that researchers most commonly involve users as consultants (low depth of participation) and mostly in one stage of the research process (narrow scope of participation); very few involve users as partners throughout the research process [22]. These results can be seen in the light of Arnstein’s ladder of participation [44, 45], which illustrates that consultation is a form of tokenism, meaning a symbolic effort to be inclusive to members whose voices are seldom heard, rather than genuine participation with equal power levels. Yet, such hierarchical beliefs implying that people are either included or excluded in decision-making processes have been criticized as ignoring important aspects of user involvement. Hence, the aim and scope of user involvement depends on who the participating users are, and the methods used to involve and secure active involvement over time must be adopted to fit their specific needs and prerequisites [46].

### Motivations for user involvement

Despite the fact that the European Commission promotes partnerships [5] and that research funders increasingly ask for user involvement in their calls for research proposals, just 16% of the researchers with previous experience of involving users in our study gave the requirement of research funders as a motivation. They were rather motived to involve users in their projects because of the added value it brought, such as to ensure that the research was relevant to the target population, strengthen the validity and trustworthiness of the results and strengthen the possibilities for implementation. In a qualitative study, Thompson [27] explored health researchers’ attitudes towards user involvement and found that user involvement was motivated by ethical priorities, which was not shown in our study. This divergence might be due to how the statement was formulated (i.e. ethical imperative) as well as the different study designs (i.e. qualitative versus quantitative). Still, although our participants did not get a chance to talk and reflect upon ethical and moral rationales for involvement, the free-text answers provide information that can be used to optimize future surveys to capture ethical motivations in a more qualified way. We did not find differences in motivations for involving users based on sex or career stage, which is not surprising given the low sample size. However, future studies should explore whether there are different motives depending on the research interest and background of the researchers. For example, in comparison with lab-based researchers, researchers from health and social sciences might hold different rationales for involving users in their research.

### Benefits and challenges of user involvement

Most of the benefits identified in this study have been described in earlier works [22, 27]. Yet, in addition to previous findings, our study demonstrates that user involvement in the context of research on ageing and health may also be beneficial in terms of redesigning interventions so they may work in a diversity of contexts and how the reciprocal relationship between users and researchers can enhance the person-centredness of those interventions. Such knowledge is important as user involvement may help to battle challenges related to the provision of effective and person-centred healthcare [47]. Yet, to find the answers needed to deliver cost effective local healthcare and social services of high quality that are user friendly, and support older adult’s well-being, researchers and providers need to involve a broader range of users, not only those receiving the care.

The researchers in our study had involved healthcare and/or social service professionals to a higher degree than other categories of users, and many stated that involving users was motivated by the quest to strengthen the possibilities for implementation. From a user perspective, Laustsen and colleagues [48] found that healthcare professionals involved to co-develop and implement an intervention used their practical experience and knowledge about clinical prerequisites to ensure that the intervention was designed in a way that was useful and improved quality. They also found, in another study, that researchers who had involved healthcare professionals in their research believed that doing so was important as it may lead to professional role development, foster a holistic perspective and improve healthcare for older adults [49]. Yet, user involvement in research can be a challenging task [49–51]. For example, in our study the researchers who had involved professionals in research processes provided examples of concerns regarding scientific quality and how recruitment and continued user involvement were challenged by the lack of organizational support and resources and by time constraints. Thus, when involving professionals, it is important to be knowledgeable of and adapt the research to preconditions such as environment, available resources and organizational conditions.

In research on ageing and health, users commonly have specific needs and prerequisites. For example, those caring for an older partner or spouse may not prioritize being involved in research due to their lack of time [52]. In addition to previously reported challenges such as resource demands and power issues, our findings suggest that involvement of older adults and persons with specific needs requires a more accommodating and attentive methodological approach. Hence, training and guidance might be needed for researchers to become more knowledgeable about how to best involve users with diverse needs in different research activities [41]. For example, the challenges that the researchers in our study described show a need to support frail older adults to articulate their views in involvement activities and provide information that can be easily understood, which illustrates the need for researchers to have advanced skills. Schilling and Gerhardus [22] found that involving older adults with age-related conditions in research presents challenges that need to be considered, such as communication needs, mobility restrictions, timing of involvement, lack of continuity, difficulties in relationships and limited confidence to contribute. The National Institute for Health Research [53] has put forward publications that can be used as a support for researchers as well as for users, but evidence-based guidelines focusing specifically on the methodological challenges ageing and health researchers may face are currently lacking. In addition, involving users may add additional pressure in a competitive academic environment [28], which makes it important to also consider organizational support. Thus, an extended depth, breadth and scope of user involvement [54] in research is not always preferable but is dependent on the specific context.

### Attitudes towards user involvement in research

Interestingly, the findings suggest that there is no consensus at all (almost evenly divided) among ageing and health researchers regarding whether users should be actively involved in publicly funded research. Based on these findings, future studies should investigate when and under which circumstances user involvement is good or bad. Furthermore, there seems to be a controversy in attitudes between older adults in the general population and researchers, as a larger proportion of older adults than researchers reported that users should be involved (90% vs 47%) and that involvement is a prerequisite for the research to lead to changes in society (88% vs 65%). These results could serve as an incentive for researchers to be more responsive to research initiatives and needs that are expressed by older adults in the general population.

### Methodological considerations

The strategy we used to recruit ageing and health researchers may be seen as a limitation, but in Sweden there is no national registry or other means to define and reach this population. Accordingly, it is not possible to estimate how many ageing and health researchers there are in Sweden, which implies that the findings cannot be extrapolated beyond the participants. Still, using the SWEAH network and the member list of the SGS, we managed to recruit researchers with different disciplinary backgrounds from numerous different universities. In addition, we do not know anything about the researchers who did not respond; it may well be that many of them lacked previous experience or had low interest or negative attitudes towards user involvement. As to the general population sample of older adults, there are issues related to representativeness (e.g. health status, education). Still, as previously reported by Frögren and colleagues [34], 11.1% of our sample was at risk of frailty; hence, our study sample included responses reflecting the perspectives of some older adults with poor health. In comparison with the general population of older adults in Sweden, our sample had a higher level of education, and the youngest (60–64 years) and oldest (85 years and older) were underrepresented [34]. The results of a study focusing on the sample of older adults in the panel study showed that those with a higher level of education were more willing to be actively involved in research regardless of whether they had been involved in research before [34]. Hence, the older adults in our study may to some degree be seen as professional laypersons who are familiar with the academic language including the terms used when talking about user participation in research. Future research should explore how to recruit and involve older adults with lower education as well as those over the age of 85, as they may have conflicting opinions and interests different from those presented in this study.

Another potential limitation is the differences in the two questionnaires used. That is, the researchers answered questions on user involvement in research as a broad phenomenon involving different categories of users and from their perspective of being researchers, while the older adults answered questions from their perspective as members of the public being involved in research. This was due to the outcome of the user forum in the refinement of survey content and may have had an impact on the comparisons. Yet, this study is the first to explore and compare ageing and health researchers’ and older adults’ attitudes towards user involvement, which makes the study a valuable contribution and inspiration for future research.

## Conclusions

This study reveals significant differences in attitudes towards user involvement between older adults in the general population and researchers in ageing and health. Ageing and health researchers with previous experience of user involvement have involved different categories of users in their research and do so because they think it makes the research more relevant and of better quality. While several challenges were identified, training for researchers and users, institutional support and necessary resources from funders could alleviate many of these challenges. Yet, the low response rates and issues related to representativeness should be taken into account when interpreting the findings from this explorative study. Further research with comparable larger samples combined with qualitative methods allowing researchers and users to describe their experiences is needed to confirm and understand the possible consequences such controversy might have and how to solve them. Expanding this knowledge may promote research partnerships as well as inform researchers, policy-makers and funding agencies about how to increase the quality of research conducted with or by users in the future.

## Data Availability

The datasets used and/or analysed during the current study are accessible from the corresponding author on reasonable request.
